# Analysis of Faecal Zonulin and Calprotectin Concentrations in Healthy Children During the First Two Years of Life. An Observational Prospective Cohort Study

**DOI:** 10.3390/jcm9030777

**Published:** 2020-03-12

**Authors:** Beata Łoniewska, Karolina Adamek, Dagmara Węgrzyn, Mariusz Kaczmarczyk, Karolina Skonieczna-Żydecka, Jeremy Clark, Grażyna Adler, Joanna Tousty, Izabela Uzar, Piotr Tousty, Igor Łoniewski

**Affiliations:** 1 Department of Neonatal Diseases, Pomeranian Medical University, Szczecin 70-111, Poland; karolaadamek1@gmail.com (K.A.); dagpak@poczta.onet.pl (D.W.); joasia.tousty@gmail.com (J.T.); 2 Department of Clinical and Molecular Biochemistry, Pomeranian Medical University, Szczecin 70-111, Poland; mariush@pum.edu.pl (M.K.); jeremyclarkbio@gmail.com (J.C.); 3 Department of Human Nutrition and Metabolomics, Pomeranian Medical University, Szczecin 71-460, Poland; karzyd@pum.edu.pl (K.S.-Ż.); sanprobi@sanprobi.pl (I.Ł.); 4 Department of Studies in Anthropogenetics and Biogerontology, Pomeranian Medical University, Szczecin 71-210, Poland; grazyna.adler@pum.edu.pl; 5 Department of General Pharmacology and Pharmacoeconomics, Pomeranian Medical University, Szczecin 71-230, Poland; uzari@wp.pl; 6 Department of Obstetrics and Gynecology, Pomeranian Medical University, Szczecin 70-111, Poland; piotr.toscik@gmail.com

**Keywords:** zonulin, calprotectin, children, antibiotic, Body Mass Index (BMI), body mass, breast feeding, caesarean section, birth, gut barrier

## Abstract

Factors affecting the intestinal-barrier permeability of newborns, such as body mass index (BMI), nutrition and antibiotics, are assumed to affect intestinal-barrier permeability in the first two years of life. This study assessed 100 healthy, full-term newborns to 24 months old. Faecal zonulin/calprotectin concentrations were measured at 1, 6, 12, 24 months as gut-permeability markers. Zonulin concentrations increased between 1 and 12 months (medians: 114.41, 223.7 ng/mL; respectively), whereas calprotectin concentrations decreased between one and six months (medians: 149. 29, 109.28 µg/mL); both then stabilized (24 months: 256.9 ng/mL zonulin; 59.5 µg/mL calprotectin). In individual children, high levels at one month gave high levels at older ages (correlations: calprotectin: between 1 and 6 or 12 months: correlation coefficient (*R)* = 0.33, statistical significance *(p)* = 0.0095; *R* = 0.28, *p* = 0.032; zonulin: between 1 and 24 months: *R* = 0.32; *p* = 0.022, respectively). Parameters which gave marker increases: antibiotics during pregnancy (calprotectin; six months: by 80%, p = 0.038; 12 months: by 48%, *p* = 0.028); vaginal birth (calprotectin: 6 months: by 140%, *p* = 0.005); and > 5.7 pregnancy-BMI increase (zonulin: 12 months: by 74%, *p* = 0.049). Conclusions: “Closure of the intestines” is spread over time and begins between the sixth and twelfth month of life. Antibiotic therapy, BMI increase > 5.7 during pregnancy and vaginal birth are associated with increased intestinal permeability during the first two years of life. Stool zonulin and calprotectin concentrations were much higher compared with previous measurements at older ages; clinical interpretation and validation are needed (no health associations found).

## 1. Introduction

The developing gastrointestinal system has important roles in the creation of a barrier to infectious materials as well as having a nutritional role [1]. Intestinal permeability must be regulated to promote newborn growth but also to resist types of newborn diseases [2]. Individual differences in permeability might arise due to differences in gestational age, clinical status, diet patterns and age of assessment [3]. Increased permeability can have beneficial effects, such as increased nutrient uptake or development of systemic immunological tolerance [4], but has disadvantages, such as increased uptake of microorganisms and foreign antigens, leading to risk of development of infection, inflammation and systemic hypersensitivity [4,5]. At birth, intestinal permeability is high and then drops sharply after delivery, which leads to a process known as “gut closure” [2], a phenomenon which is poorly understood.

In humans, growth factors, hormones, breast milk and changes in the thickness and viscosity of the mucus gel layer play a role in this process [2,6,7]. “Gut closure” is associated with two physiological mechanisms [8]: (1) the movement of macromolecules from the lumen through paracellular spaces into the blood stream, and (2) the active transport of Immunoglobulin G (IgG) and immune complexes (ICs) from breast milk to the submucosa. Macromolecule intestinal permeability can be assessed by non-invasive methods (e.g., lactulose/mannitol test) and recently stool zonulin and calprotectin have been identified as markers of intestinal barrier development (with different kinetics concerning intestinal permeability) in healthy newborns.

Zonulin is an analogue of the cholera comma toxin (ZOT, zonula occludens toxin) [9,10] and has been shown to be important in the regulation of paracellular transport in the intestinal lumen [11,12,13]. Zonulin is a protein with a molecular weight of 47–65 kDa, which is synthesized in the liver and epithelial cells and can be isolated from multi-protein membrane complexes (claudin-occludin-guanylate kinase-like proteins ZO-1, ZO-2, and ZO-3), forming tight joints (TJ) on the apical surface of intestinal epithelium [14]. The similarity of zonulin to the precursor of haptoglobin-2 resulted in it obtaining the alternative name of pre-HP2 (prehaptoglobin-2) [15]. Its reversible effects on TJ tightness makes it one of the main factors guaranteeing the proper functioning of the intestinal barrier [16,17].

Increased zonulin concentrations have been correlated with increased intestinal permeability [18]. In patients suffering from inflammatory diseases (type 2 diabetes, celiac disease, obesity) or autoimmune diseases (Crohn’s disease, type 1 diabetes), there may be an increase in zonulin levels [14,16,19,20,21,22].

Calprotectin is secreted from stimulated neutrophils, eosinophils, and monocytes and is also expressed in some mucosal epithelial cells. Calprotectin is stable in stool and is eliminated intact in the faeces. Human calprotectin is created by a 24 kDa dimer from the protein monomers: S100A8 (10,835 Da) and S100A9 (13,242 Da) and this complex constitutes up to 60% of the soluble proteins contained in the cytosol of human neutrophils [23,24].

The calprotectin concentrations in faeces (faecal calprotectin) provide one marker for inflammatory bowel diseases, and as such, it is easy to measure and is sensitive. Elevated calprotectin levels have been described in both adults and children with inflammatory bowel diseases, such as Crohn’s disease and ulcerative colitis, and can be used to assess the severity of inflammation in these patients [24]. It has been found that faecal calprotectin levels can indicate cow-milk allergy and atopic disease, as well as gastrointestinal disorders [25]. The diagnostic value of calprotectin in infancy is also of growing interest [26].

The higher content of stool calprotectin in infancy may result from increased intestinal permeability, the formation of intestinal microbiota and/or response to food antigens. Increased faecal calprotectin concentrations correlate with the severity of inflammatory processes in inflammatory bowel diseases [27,28], but can also occur in healthy newborns [26].

In our clinic, a previous study [29] found that maternal-foetal factors, such as caesarean section, antibiotic therapy during pregnancy and body mass index (BMI) increase > 5.7 during pregnancy may have impacted on increases in intestinal barrier permeability in newborn children up to seven days of age.

The purpose of this study was to evaluate stool zonulin and calprotectin concentrations as markers of intestinal barrier permeability in the first two years of life (one month to 24 months) in children from the cohort previously described [29] to verify two hypotheses. The first is that factors that affect intestinal barrier permeability in the neonatal period (until the seventh day of life) correlate with intestinal-barrier permeability factors in the first two years; the second, a claim that factors, such as children’s body mass, type of feeding (breast or formula) and antibiotic therapy can affect intestinal-barrier permeability factors in children.

## 2. Materials and Methods

### 2.1. Subject Characteristics

The presented work was created as a continuation of research on intestinal barrier in a cohort of newborns. In the previous stage, the intestinal-barrier status was assessed in mothers before delivery and in newborns in the first week of life. In the presented second stage of the study, based on the same cohort, the state of the intestinal barrier in children between one and twenty-four months of age was assessed [29].

**Figure 1:**
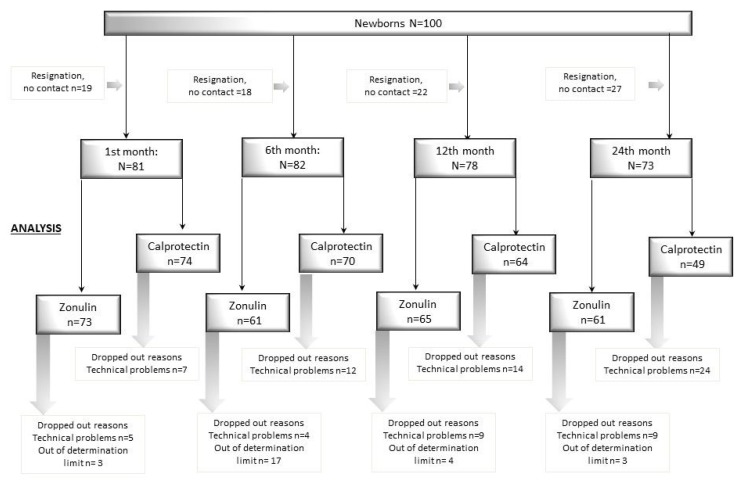
Study flow chart. Out of determination limit: see Materials and Methods. Technical problems: too little material.

The study included 100 healthy, full-term newborns born in the Department of Obstetrics, Gynaecology and Neonatology at the Independent Public Clinical Hospital No. 2 of the Pomeranian Medical University in Szczecin during the period from March 2015 to April 2016. (Figure 1 flow chart).

The condition of each newborn after birth was assessed according to the Apgar scale and on the basis of the results of umbilical-cord blood-gas assessments. Inclusion criteria: All qualified newborns after birth were rated above 7 points on the Apgar scale after 3 min of life, and the pH of the umbilical cord blood was > 7.2. Interleukin 6 (IL6) and C-reactive protein (CRP) levels were also evaluated in umbilical cord blood for the early identification of newborns with congenital infections. Newborns were included in the study with IL6 below 30 pg/mL or CRP below 5 mg/L and with no clinical signs of congenital infection.

The study excluded children born to mothers with autoimmune diseases (including type 1 diabetes), pregnancies complicated by gestational diabetes mellitus (GDM), with HELLP syndrome (Haemolytic anaemia, Elevated Liver enzymes, Low Platelet count), prematurely born (before 37 weeks of gestation), in asphyxia (Apgar ≤ 7 after 3 min of age), with congenital infection (clinical signs of infection, increased CRP, IL6) or with birth defects or those not conforming to the inclusion criteria.

All newborns were solely breastfed in the first week of life and discharged home. At 1, 6, 12 and 24 months old, the authors contacted the parents of children to perform interviews and take stool samples in order to determine zonulin and calprotectin concentrations. The interview included data on a child’s health (infections, antibiotic therapy), diet and weight. The children were healthy and did not take antibiotics at the time the sample was taken

### 2.2. Ethical Information

Consent to perform the study in the Neonatal Pathology Clinic was obtained from the Bioethics Committee of the Pomeranian Medical University with Resolution No. KB-0012/55/14 of 30.06.2014; the study followed the Declaration of Helsinki (2013). The written informed consent was obtained from participating patients.

### 2.3. Enzyme Immunoassays

Stools collected at 1, 6, 12 and 24 months old were used for zonulin and calprotectin determinations. Stool was collected from a diaper using a standardized biological material collection kit (Stool Sample Application System (SAS); Immundiagnostik, Bensheim, Germany). The stool was collected by previously trained parents according to an established procedure and stored in a home refrigerator (for a maximum of eight hours) until collected by a researcher. Transport to the laboratory lasted up to 60 min, at +6 to +8 °C. The stool was then frozen to −20 °C, for no longer than 3 months, until determinations were made.

Extracts were prepared using 15 mg of thawed faeces at room temperature. To this 0.75 mL extraction buffer was added and the resulting solution (dilution 1:50) was stored at 4 °C (for up to 7 days) until the determinations. After analysis according to manufacturer protocols, absorbance was measured at 450 nm against a reference wave of 620 nm. The zonulin and calprotectin concentrations were estimated based on a four-parameter logistic regression algorithm, using the software available at http://www.elisaanalysis.com/ap.

Faecal zonulin and calprotectin concentrations were determined by enzyme immunoassay using commercial kits for enzyme-linked immunosorbent assays (ELISAs; Immunodiagnostik). The zonulin measurements were conducted in the Independent Gerontobiology Laboratory and Calprotectin was measured at the Department of Biochemistry and Human Nutrition, both at the Pomeranian Medical University. As the dilution factor for zonulin was 50, and for calprotectin 2500, and the highest concentrations of standards were 16 ng/mL and 840 ng/mL, respectively—we determined that the highest concentrations we were able to measure were 800 ng/mL and 2100 ug/mL, respectively. Results higher than these limits were excluded from the analysis.

### 2.4. Statistical Analyses

Conformability of variables with normal distributions was tested using the Kolmogorov–Smirnov test. Most variables were not normally distributed, so non-parametric tests were used: the Mann–Whitney or Kruskal–Wallis (along with Conover’s post hoc analysis) for unpaired data, and the Wilcoxon signed rank test for paired data. Spearman’s correlation coefficients were also determined to analyse correlations between concentrations at different ages. For effect sizes the following were used: for Mann–Whitney and Wilcoxon signed rank tests: *r* = (Z/√n), where z = normalized U value, *n* = total number of observations; for Kruskal–Wallis tests: epsilon-squared = ($\frac{H}{(n^{2}-1)/\left(n+1\right)}$), where H = H test statistic, *n* = total number of observations. All calculations were made using Medcalc (ver. 19.1.5, Ostend, Belgium) and Microsoft Excel (ver. Office 2010, standard edition, Redmond, WA, USA). A 5% significance level was used.

## 3. Results

### 3.1. Population

The children were from single pregnancies and were born between 37 and 41 weeks of gestation. The characteristics of the study group are presented in Table 1 and Table 2.

### 3.2. Zonulin Concentrations

**Figure 2:**
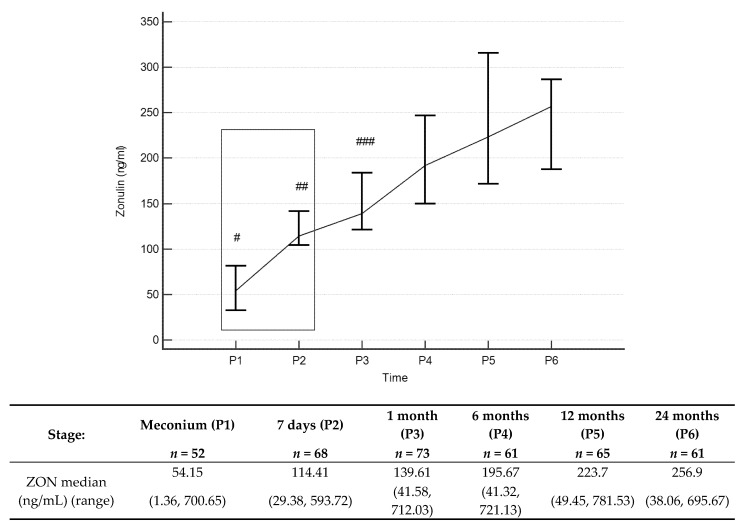
Zonulin (ZON) concentrations (median) over time. Legend: Error bars represent 95% confidence intervals for medians. For Kruskall–Wallis test *p* < 0.000001. Conover’s post-hoc analysis: # *p* < 0.05 P1 vs. P2, P3, P4, P5 and P6; ## *p* < 0.05 P2 vs. P1, P4, P5 and P6; ### *p* < 0.05 P3 vs. P1, P5 and P6. From Wilcoxon paired test regarding time: # *p* < 0.05 P1 vs. P2, P3, P4, P5 and -P6; ## *p* < 0.05 P2 vs. P4, P5 andP6; ### *p* < 0.05 P3 vs. P5 and P6. To better illustrate changes in ZON levels in the first two years of life, ZON levels in meconium, and stool from day 7, are marked with a box (these results were presented in our previous work [29]).

The zonulin concentrations (Figure 2 and Supplementary Table S1) in stool increased throughout the entire observation period, with statistically significant differences occurring between values obtained in the first, 12th and 24th month of age. Median stool zonulin concentrations at 12 and 24 months were 223.7 ng/mL and 256.9 ng/mL, respectively. Zonulin concentrations at 1 month positively correlated with zonulin concentrations at 24 months (*p* = 0.022, *R* = 0.32), showing that for an individual newborn, a high level at one month tended to give a high level at 24 months, and correspondingly for low values. 

### 3.3. Calprotectin Concentrations 

**Figure 3:**
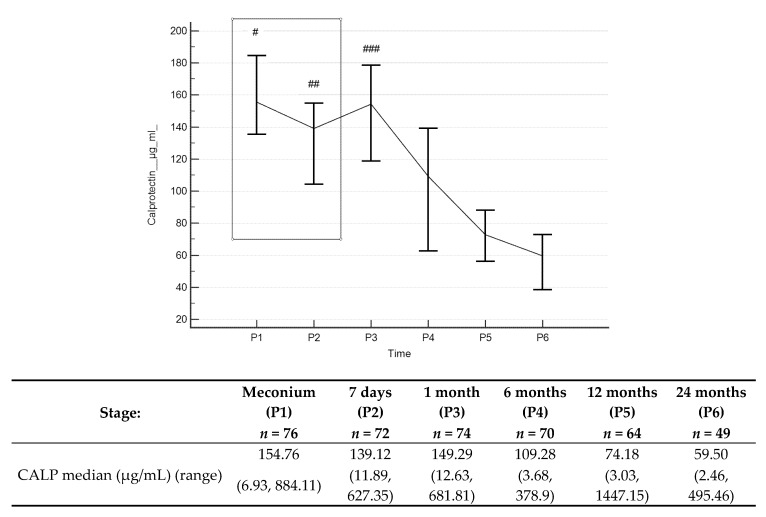
Calprotectin (CALP) concentrations (median) over time. **Legend:** Error bars represent 95% confidence intervals for medians. For Kruskall–Wallis test *p* < 0.000001. Conover’s post-hoc analysis: # *p* < 0.05 P1 vs. P4, P5 and P6; ## *p* < 0.05 P2 vs. P4, P5 and P6; ### *p* < 0.05 P3 vs. P4, P5 and P6. For Wilcoxon paired tests comparing times: # p<0.05 P1 vs. P4, P5 and P6; ## *p* < 0.05 P2 vs. P5 and P6; ### *p* < 0.05 P3 vs. P4, P5 and P6. To better illustrate changes in CALP levels in the first two years of life, CALP levels in meconium, and stool from day 7, are marked with a box (these results were presented in our previous work [29]).

Stool calprotectin concentrations (Figure 3 and Supplementary Table S2) decreased throughout the entire observation period, with statistically significant differences occurring between values obtained in the first month, and 6, 12 and 24 months of age. Median stool calprotectin concentrations at 12 and 24 months were 74.18 µg/mL and 59.5 µg/mL, respectively. There were positive correlations between calprotectin concentrations at 1 month and concentrations at 6 months (*R* = 0.33, *p* = 0.0095) or 12 months old (*R* = 0.28, *p* = 0.0318), showing that for an individual newborn, a high level at one month tended to have higher level at older ages, and correspondingly for low values. 

### 3.4. Effects of Antibiotic Therapy During Pregnancy

**Figure 4:**
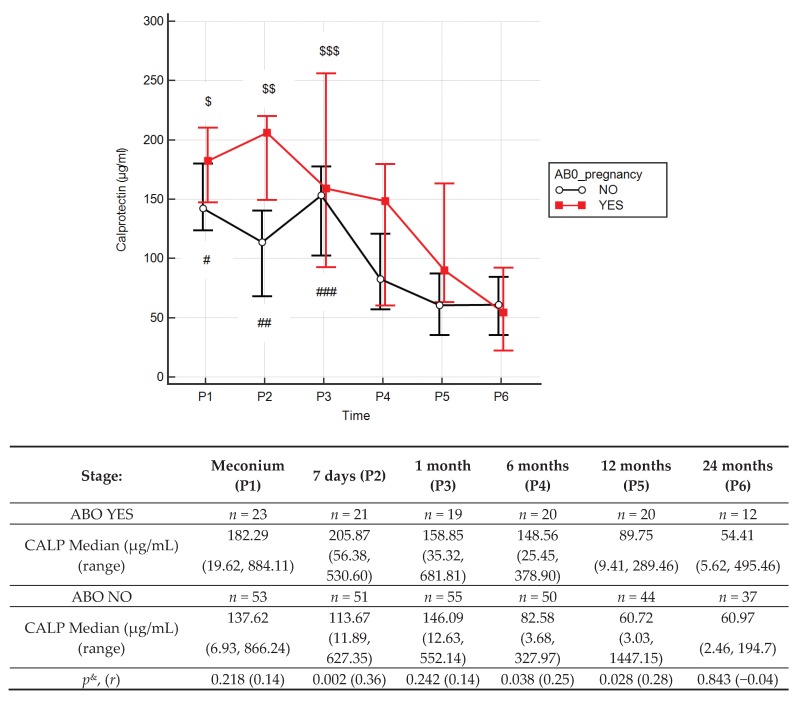
Calprotectin (CALP) concentrations (median) in children over time with (red line) or without (black line) antibiotic therapy during pregnancy (ABO). Legend: Error bars represent 95% confidence intervals for medians. *p*^&^ = Mann–Whitney test comparing effects of antibiotics (ABO), *r* = effect size. Wilcoxon paired tests comparing times, # (No ABO), $ (ABO): *p* < 0.05 P1 vs. P4, P5 and P6; ##, $$: *p* < 0.05 P2 vs. P5 and P6; ###, $$$: *p* < 0.05 P3 vs. P4, P5 and P6. To better illustrate changes in CALP levels in the first two years of life, CALP levels in meconium, and stool from day 7, are marked with a box (these results were presented in our previous work [29]).

The use of antibiotics in pregnant women did not affect the zonulin concentrations in children (Supplementary Table S3). Stool calprotectin concentrations (Figure 4) in children of mothers treated with antibiotics during pregnancy were significantly higher at 6 (by 79.9%) and 12 (by 47.8%) months old (*p* = 0.038; *p* = 0.028) than without antibiotics.

### 3.5. Effects of Type of Delivery

**Figure 5:**
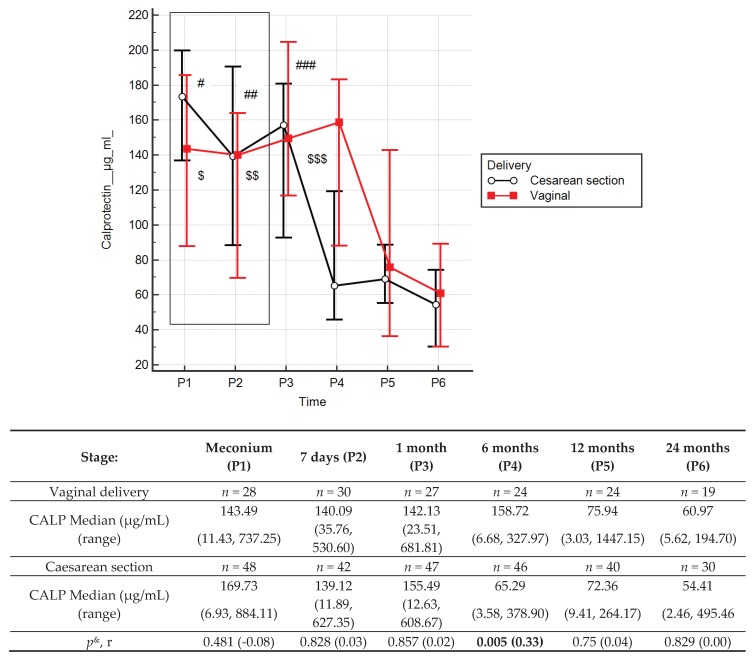
Calprotectin (CALP) concentrations (median) over time with delivery type: caesarean section (black line) or vaginal birth (red line). Legend: Error bars represent 95% confidence intervals for medians. *p*^&^ = Mann-Whitney test comparing effects of delivery type, *r* = effect size. Wilcoxon paired tests comparing times, # (Caesarean section), $ (Vaginal delivery): #, $ *p* < 0.05 P1 vs. P4, P5 and P6; ##, $$ *p* < 0.05 P2 vs. P4, P5 and P6; ###, $$$ *p* < 0.05 P3 vs. P4, P5 and P6. To better illustrate changes in CALP levels in the first two years of life, CALP levels in meconium, and stool from day 7, are marked with a box (these results were presented in our previous work [29]).

The type of delivery had no effect on zonulin levels in the stool of children from 1 to 24 months of age (Supplementary Table S4). In children born vaginally, the calprotectin concentrations at 6 months were 143% higher compared to children born via caesarean section (158.72 vs. 65.29; *r* = 0.33, *p* = 0.005) (Figure 5). There were no differences in the stool zonulin and calprotectin concentrations between children treated with antibiotics or not during delivery (Supplementary Table S5). 

### 3.6. Effects of BMI Increase in Pregnant Women and Body Mass Up to Two Years Old

Children of mothers who had an increase in BMI > 5.7 during pregnancy showed a 73.7% increase in zonulin concentrations at 12 months of age (*p* = 0.049, *r* = 0.25). The increase in BMI in pregnant mothers had no effect on the stool calprotectin concentrations of children between 1 and 24 months of age (Supplementary Table S6). Birth weight and later weight of children had no effect on the stool zonulin and calprotectin concentrations (Supplementary Table S7–S10).

### 3.7. Effects of Antibiotic Therapy and of Type of Feeding in Children Up to 2 Years Old

In children who received antibiotics between the first and fourth week of life, the concentrations of calprotectin in the stool were 49% lower (*p* = 0.02, *r* = 0.24) compared to newborns who had not received antibiotics during this period. In 12-month-old children fed naturally, calprotectin concentrations were 42.8% higher compared to results obtained in formula-fed children (*p* = 0.048, *r* = −0.25); however, the sample size (*n* < 10) was too small to draw conclusions (Supplementary Table S11, Supplementary Figure S1, S2).

## 4. Discussion

This is the first study in which the effects of maternal-foetal and environmental factors on concentrations of zonulin and calprotectin in children’s stool between the first month and the second year of life has been examined.

The study of zonulin and calprotectin measurements allow non-invasive assessment of the functional state of the intestinal barrier, with zonulin considered to be a marker for increased barrier permeability, and calprotectin a marker for intestinal inflammation, which may also be associated with increased permeability. 

In humans, intestinal permeability to macromolecules is very high shortly after birth. In the postpartum period intestinal permeability decreases: a process which is referred to as “gut closure” [2,30]. This decrease also continues gradually over the next few months or maybe years [31,32]. 

It is currently unknown whether the prolonged second phase, with a slow reduction in intestinal permeability plays an important physiological role. Studies that measured permeability using lactulose/mannitol ratios up to two weeks old showed enhanced prolongation in newborns fed artificially compared with those breast-fed [30,33], but then similar gut permeability at one month old. Veereman-Wauters [7] reported that factors, such as gestational age and enteral nutrition, can play a role in intestinal maturation. It has been suggested that the presence of intestinal microbiota may facilitate intestinal development [34]. As there are many different factors in the early stages of life that could affect permeability, further research is required, especially as it is possible that newborn permeability may affect metabolic health later in life.

The zonulin concentrations observed in the present study at one month old were significantly different from those at 12 and 24 months old. It is also noteworthy that individuals with high permeability factors at one month tended to have high factors at older ages and correspondingly for low values (shown by positive correlations). It can therefore be assumed that for most individuals the paracellular permeability of the intestinal epithelium stabilizes sometime between 1 and 12 months old, after decreasing from a peak. Taking into consideration median values, high standard deviations of the obtained results and the borderline statistical significance (zonulin concentrations: 6th month vs. 1st month—*p* = 0,064 and 24th month vs. 6th month—*p* = 0,058), it can be assumed that stabilisation of intestinal permeability occurs between sixth and twelfth month of life. Of course, based on the results obtained, it is impossible to determine the specific moment of “intestinal closure”, which, moreover, must be a process that takes place over some time.

It should be emphasised that in healthy children, the zonulin concentrations in the stool remain at a fairly high level up to 24 months of age, exceeding 200 ng/mL. We have not found other literature giving data on zonulin concentrations in children’s stool up to two years old (only zonulin concentrations in children’s blood have been given). Tarko et al. [35] found significantly higher zonulin blood concentrations in children with abdominal wall defects and rotavirus infection. In another study, blood zonulin levels in children born before 28 weeks of pregnancy were lower compared to a control group (note that the zonulin levels were not given) [36]. Therefore, we can hypothesise that increased zonulin levels show present or previously increased intestinal permeability.

An important problem that may affect the results of zonulin measurements may be the particular ELISA method used for measurements. ELISA is commonly used in laboratories to assess the integrity of the intestinal barrier. Scheffler [37] suggested that a serum zonulin (pre-HP2) test (Immundiagnostik ELISA kit) may simultaneously detect a variety of proteins that resemble zonulin structurally and perhaps functionally. This would mean the existence of a zonulin protein family instead of one protein structure regulating intestinal permeability. This hypothesis has already been presented in earlier studies [38]. However, the authors emphasise the need for additional research to define the fundamental proteins (zonulin, properdin and/or other structurally similar proteins) measured in the commonly available enzyme-linked ELISAs (and whether the components detected differ from test to test).

Current literature gives different values for stool zonulin concentrations. Malíčková et al. [39], based on the recommendations of the ELISA manufacturer-OrgenTec, Germany, indicated normal zonulin concentrations of 61 ng/mL ± 46 ng/mL. On the other hand, the cut-off upper normal limit for adults given by Lamprecht et al. was a zonulin concentration value of 30 ng/mL [40]. The results observed in our work are much higher than this cut-off point. It should be emphasised that zonulin levels above 30 ng/mL and 100 ng/mL were observed in approximately 92% and 8% of children at 12 months of age and 90% and 10% of children at 24 months of age, respectively. Our results suggest setting a different cut-off point for stool zonulin levels for children than in adults. They also indicate the need for further research in this area.

Faecal calprotectin concentrations depend on the number of neutrophils migrating across the gastrointestinal mucosa. This may be associated with inflammatory diseases of the gastrointestinal tract, and in infancy, higher values may result from increased intestinal permeability, response to food antigens, as well as colonisation of the intestine by bacteria. The increased calprotectin concentrations observed in our study at one month old, above those at delivery (meconium), then significantly started to reduce until six months old and stabilised until the end of the study period. These results are consistent with observations from other authors. In a group of healthy, full-term newborns, as well as in premature newborns, less than three months old, increased stool calprotectin values, similar to those observed in children and adults with inflammatory bowel disease (IBDs), have been found [41,42]. Baldassarre [43] described a small but significant increase in calprotectin concentrations at day seven compared to concentrations at day three for newborns born at term. Subsequent stool calprotectin values remained stable during the first month of life. In a study from Lee et al. [44], the average concentrations of calprotectin decreased with time from 322 μg/g (from birth to two months) to 197 μg/g (at two to four months of age) to reach a mean concentration of 111 μg/g at the age of four to six months. In another work, Li et al. [45] studied stool calprotectin concentrations in a population of 288 healthy Chinese children aged one to eighteen months. The median concentration was 174 μg/g (range: 6.0 to 1097.7 μg/g). Higher calprotectin values have been found in other studies performed in newborns compared to older children [46,47]. According to previous results [43,44,48], data from Chinese authors confirm that newborns in the first months of life have higher calprotectin concentrations than healthy older children. Median values measured were 375 μg/g (range: 77 to 962 μg/g) at the age of one to three months, 218 μg/g (range: 53 to 621 μg/g) at three to six months old, after which the concentrations decreased to approximately 100 μg/g at the age of 6 to 18 months. In our study, the ranges of measured calprotectin concentrations in stool at successive ages up to 24 months are given in Figure 3 and Supplementary Table S2, which may be useful in clinical practice to determine normal ranges during this period. 

High levels of stool calprotectin in the first months of life seem to be a natural phenomenon and is most likely associated with the continuing development of the gastrointestinal tract and the lack of maturation of the immune system [48,49]. High levels of stool calprotectin may reflect increased transepithelial migration of granulocytes or newly formed macrophages due to immaturity of the intestinal mucosa [18,50]. A possible physiological reason for increased calprotectin concentrations is that the functional immaturity of the intestinal barrier includes low levels of antibodies, which are unable to provide control over intestinal microbiota and therefore granulocytes fill this gap in protection.

High levels of calprotectin in the first few days may follow increased intestinal permeability, response to food antigens and intestinal colonisation with commensal microorganisms, which protect against infectious pathogens and prevent interaction between host cells and pathogens [42,51,52,53]. It has been found that intestinal colonisation in the first weeks of life and chemotactic compounds (such as N-formyl-methionyl-leucyl-phenylalanine) lead to an increase in calprotectin concentrations [54] by stimulating transepithelial migration of granulocytes during the development of food tolerance and regulation of microbiota [55]. The third possible reason why calprotectin levels in the stool might be elevated in the first month of life is from subclinical, physiological gastrointestinal inflammation, also associated with the migration of granulocytes into the intestinal lumen [49,56].

Calprotectin, due to its numerous biological activities such as fungicidal, bactericidal and immunomodulatory properties, may play roles in the intestinal environment of healthy newborns by providing beneficial effects on the newborn’s immune system [42,45]. 

For all of the above reasons, the determination of this marker in stool provides a simple and non-invasive test that can be useful in paediatric diagnostics. However, measurement ranges should be determined at different times after birth, and the present study is a contribution to this research. The test description provided by the ELISA manufacturer (Bühlmann Laboratories AG, Schönenbuch, Switzerland) presents the following threshold values for calprotectin concentrations in stool: for healthy children from 4 to 17 years of age < 50 μg/g; in mild organic disease: between 50 and 200 μg/g; in active organic disease: > 200 μg/g.

However, the above thresholds do not apply to children aged 1 to 24 months. Olafsdottir [48] found much higher mean stool calprotectin concentrations in healthy newborns than in healthy children over the age of one year (278 μg/g vs. 40 μg/g). Hestvik et al. [57] showed that the calprotectin concentrations in healthy children was 249 μg/g under the age of one year, 75 μg/g at the age of 1–4 years and 28 μg/g at the age of 4–12 years. Berni et al. [58] reported that the mean calprotectin concentration was 28 μg/g in healthy children aged 13 to 216 months. Ezri et al. [59] showed that stool calprotectin concentrations varied depending on the age of the child, with a normal upper cut-off point in children under one year of < 350 μg/g, in older children < 275 μg/g and in adults < 50 μg/g.

Although it is known that calprotectin concentrations vary with age, in children under 4 years of age, 77 µg/g [60] has been proposed as the upper limit and 50 μg/g has become the cut-off point for patients older than 4 years [47,57,61]. Further, Peura et al. [26] proposed the following upper limits (µg/g; 90% confidence interval) for stool calprotectin concentrations in children aged 0–24 months: 0 months: 324 (274–381); 6 months: 615 (189–1057); 12 months: 136 (119–179); 24 months: 57 (57–64). In a meta-analysis of prospective studies conducted by Von Roon et al. [62] with 5983 patients, the observed sensitivity and specificity of stool calprotectin measurements for inflammatory bowel disease (IBD) and other inflammatory bowel diseases were 86% and 81%, respectively, and higher precision was achieved with a cut-off of 100 μg/g.

Our stool calprotectin results in healthy children are similar or lower than those reported in other studies [42,45,63]. The differences in calprotectin concentrations may result from the influence of genetic and environmental factors, differences in particular ELISA tests, or a combination of all these factors [64]. Stool calprotectin measurements should be interpreted cautiously in younger children, especially in the first year of life [65]. The evaluation of calprotectin as a marker of intestinal disease is controversial, as healthy newborns have large inter-individual variability [66]. In addition to using a cut-off point for calprotectin concentrations in the stool, to increase its diagnostic value it would be advisable to perform a sequence of determinations in individual patients at successive ages [61].

Kapel et al. [42], based on a review of 20 studies with 1180 premature and premature newborns, observed that stool calprotectin levels greater than about 350 μg/g may suggest the presence of gastrointestinal disease. In our study, approximately 8% of healthy children had calprotectin levels above 350 μg/g. These children had no signs of intestinal infection such as vomiting, diarrhoea, fever or other specific symptoms. Based on the presented study, we were not able to demonstrate the potential relationship of high calprotectin values obtained in the stool with the development of intestinal diseases, as this aspect would have to be assessed over a longer period with a larger sample size. Therefore, for research purposes, it will be necessary to determine calprotectin concentrations in large cohorts of children (especially under the age of 12 months). 

An interesting observation is inverse relationship between zonulin and calprotectin levels. The tight junction barrier exhibits both size and charge selectivity with two distinct routes across an intact epithelial monolayer, termed the ‘pore’ and ‘leak’ pathways [17,38]. Zonulin is one of the main factors, which secures adequate action of the “gut gateway” mechanism by reversibly influencing the tightness of TJs [17,38], influencing incorporation of proteinase-activating receptor 2 (PAR2) and epidermal growth factor receptor (EGFR), and allowing molecules exceeding size of approximately 3.5 kDa to cross the intestinal barrier [15]. Zonulin activates zonula occludens (ZO) 1 proteins and is involved in a low-capacity leak pathway permeability with a more limited selectivity [67,68]. However, in homeostasis and in less active inflammatory disease, the epithelium is generally intact and barrier function primarily reflects flux across the paracellular pore and leak pathways [69,70,71]. It means, that in newborns the crosstalk between gastrointestinal and gut associated immune system is less restrictive and allows free flow exchange of various particles. As a result of this phenomenon, increased stool zonulin concentration can be observed. Calprotectin is another marker of neutrophils migrating through the gastric and intestinal mucosa, which may be associated with inflammatory processes of the gastrointestinal tract. Calprotectin could also be viewed as a marker of intestinal permeability. However, based on our results, we assume that after birth inflammatory processes in the gut tend to decrease over time save the increased permeability of antigens shaping immune tolerance. However, this mechanism may also contribute to the pathogenesis of chronic inflammatory diseases in genetically susceptible individuals [72].

Polish researchers have indicated the usefulness of assessing zonulin concentrations as a marker of increased intestinal permeability during the development of ongoing chronic inflammation, as positive correlations between zonulin concentrations in the blood serum and body mass, body fat percentage, glucose concentration and the amount of energy supplied have been observed [73]. Increased zonulin levels were also found in pregnant women with diabetes (GDM, Gestational Diabetes Mellitus) [74]. Calprotectin is also considered a marker for obesity and metabolic disorders in both adults [75] and children [76]. The relationships between maternal weight gain during pregnancy and intestinal permeability markers in children have not previously been studied. Our study did not observe that birth weight in children over the first two years of life had an effect on zonulin and calprotectin levels in stool. Only in children whose mothers had had an increase in BMI during pregnancy of > 5.7 were the zonulin concentrations at the age of 12 months higher compared to the rest, which may indicate increased intestinal barrier permeability in this group of children.

Li et al. [45] found a negative correlation between calprotectin concentrations and anthropometric indices, showing that children achieving lower height have higher stool calprotectin concentrations, which may be evidence of increased intestinal permeability. In our previous study, we observed that pregnancy mass gain > 18 kg was associated with higher calprotectin concentrations in maternal stool and that an increase in BMI > 5.7 during pregnancy was associated with increased stool calprotectin of mothers and newborns on day seven. There was also a positive correlation between the increase in BMI in pregnancy and calprotectin concentrations in the mother’s stool [29]. If the above observations are taken into account, it seems that the body weight of mothers during pregnancy could be positively associated with intestinal permeability in children during the first two years of life. 

Our previous study [29] showed significantly higher zonulin concentrations in a group of seven-day-old newborns born by caesarean section compared to a group of vaginally born newborns. Later in life, the zonulin concentrations in the group of naturally born children did not differ from that found in children born via caesarean section. However, the calprotectin concentrations in children born vaginally at six months were significantly higher compared to those born by caesarean section, which may indicate a later “gut closure” in newborns born vaginally. These results and research from others are not conclusive. Campeotto [50] and Baldassarre [43] did not show a correlation between the type of delivery and calprotectin concentrations in newborns born at term, while Josefsson [77] showed a relationship between delivery of premature newborns by Caesarean section and stool calprotectin concentrations. Lee et al. [44] documented higher calprotectin concentrations in children born naturally compared to children born by caesarean section.

It is generally believed that the foetal digestive tract is sterile, and immediately after birth is rapidly colonized by microorganisms from the external environment [78,79]. Vaginal childbirth guarantees early contact with the mother’s microbiome and is crucial for intestinal maturation, metabolic and immunological programming, and to establish homeostasis between microorganisms and the host. Some studies have shown differences in the microbiota of newborn faeces depending on the type of delivery and the relationship between early colonisation patterns and the method of delivery [78]. Thus, contact with the “rich” microbiota of the mother during vaginal delivery hypothetically leads to colonisation of the gastrointestinal tract of newborns, which in turn stimulates the migration of immune cells and causes greater release of calprotectin into the intestinal lumen. To verify this hypothesis, it would be necessary to perform stool microbiota tests, which is currently planned and will be the subject of further research. On the basis of conducted studies, it is not possible to assume the effect of earlier “gut closure” on newborns health. It could be wise to assume that earlier “gut closure” in children delivered by caesarean section could result in altered immune tolerance due to limited contact with allergens and increased likelihood of allergy, atopy, and asthma, and reduced intestinal gut microbiome diversity [80].

Another factor that can affect the composition of microbiota, and thus can be assumed to affect the intestinal barrier, is antibiotic therapy. Antibiotic therapy during pregnancy results in higher zonulin concentrations in umbilical cord blood serum. Additionally, the use of antibiotic therapy during delivery, as in the case of Caesarean section, causes increased zonulin concentrations in the stool of seven-day-old newborns [29]. The study presented here showed that antibiotic therapy during pregnancy did not affect zonulin levels in children over one month of age, whereas calprotectin levels were significantly higher at 6 and 12 months old, which might be explained by a slower maturation of the intestinal barrier in children of mothers who underwent antibiotic therapy in pregnancy.

Approximately 40% of newborns are indirectly exposed to antibiotics during natural childbirth [79,81]. The widespread use of antibiotics in the perinatal period has short- and long-term consequences. The former include the development of antibiotic-resistant strains [82], while the long-term consequences include the occurrence of asthma and obesity [83]—diseases also associated with microbiota alterations in early life [84]. Moreover, exposure to antibiotics during the first month of life could predispose to functional gastrointestinal disorders. The link between these two conditions could be associated with an alteration of intestinal permeability [85].

The use of antibiotics by a pregnant woman affects both her own microbiome and the newborn’s microbiome, which may be a factor that affects the newborn’s immune system [86].

Despite the inconsistency with results from other studies—which have shown that stool calprotectin concentrations can be negatively correlated with antibiotic therapy in a population of newborns with very low birth weight [77]—on the other hand, in children with cystic fibrosis, antibiotic treatment was associated with insignificantly increased calprotectin concentrations [87]. The results observed in our study, which was conducted on healthy children, could suggest increased activity of neutrophils, caused for example by microbiota changes induced by antibiotic administration during pregnancy.

The above results suggest far-reaching caution when using antibiotics in pregnant women, as this may have long-term consequences for the child.

One surprise observation was that antibiotic therapy did not affect zonulin and calprotectin concentrations in children during the first two years of life. However, note that precise assessment of the timing, type and dosage of antibiotic therapy was difficult because assessments were based only on interviews with mothers. Alternative methods of data verification are difficult to carry out in Poland, because medical care is provided at several points (from the state and private healthcare systems) and there is no centralised database for child treatments). Gut microbiota analyses could shed more light on this problem. It has recently been reported that intestinal microbiota in healthy people are much more resistant to antibiotics than previously thought [88] and perhaps this also applies to the intestinal barrier and its maturation processes. Further research is needed, especially regarding the composition and function of the microbiota, to elucidate this problem.

Our results also showed no effect of feeding on intestinal permeability markers. Results from other (although not all [89,90]) studies have revealed that artificial feeding apparently favours earlier “gut closure” [45,91,92]. Calprotectin concentrations in children fed exclusively on mother’s milk compared to children fed only with artificial formulas under the age of six months seemed to confirm that breast milk stimulates the intestinal mucosa despite having simultaneous positive effects on reducing permeability [93]. Increased concentrations of calprotectin in naturally fed children may result from immunomodulatory agents in human milk, which affect the intestinal mucosa. Although calprotectin concentrations in our study were higher in breast-fed children compared to results obtained in formula-fed children, the sample size was too small to draw conclusions from our data concerning methods of feeding.

### Limitations

(1) Lack of direct intestinal permeability measurements. (2) Lack of assessment of the composition of the faecal microbiota and their metabolic function. Because of (1) and (2), we could not check a relationship between intestinal permeability, faecal microbiome and the concentrations of stool markers. (3) The need to use interview data from the mothers of children, which might have resulted in errors especially related to the assessment of the effects of antibiotic therapy. (4) No formal sample size calculations were employed for this analysis because the cohort size was defined as a continuation of a former study in which the sample size was based on numbers sufficient according to previously published studies [35,42,44,45].

## 5. Conclusions

The results of this study on intestinal permeability in children, as measured by stool zonulin and calprotectin levels, are inconsistent (zonulin levels increased and stabilized, remaining at a high level from 12 months of age, and calprotectin levels decreased and stabilized from six months). It can only be assumed that the process of “closure of the intestines” is spread over time and begins between the sixth and 12th month of life. Antibiotic therapy, and BMI increase > 5.7 during pregnancy (which are also associated with increased intestinal permeability in newborns) are associated with increased, but caesarean section (with increased zonulin levels in newborns) with decreased intestinal permeability during the first two years of life. Stool zonulin and calprotectin concentrations up to two years old were much higher than at later ages and their clinical interpretation requires caution and further research. No relationship was observed between the elevated values of these markers and any health consequences.

## Figures and Tables

**Table 1:** Group characteristics: mothers and newborns.

Characteristic			Newborns (*N* = 100)	
Gender: (% male)			54.0	
Birth weight (g)				
(mean ± SD)			3427 ± 455	
(range)			(2140, 4960)	
≤ 15th percentile			15%	
≥ 85th percentile			12%	
Characteristic			Mothers (*N* = 100)	
Vaginal childbirth			39% (*n =* 39)	
Antibiotic therapy during pregnancy			30% (*n =* 30)	
Antibiotic therapy at delivery			81% (*n =* 81)	
Body ass index (BMI) (kg/m^2^) increase during pregnancy (mean, range)			5.7 (−1.12, 11.2)	
BMI before pregnancy (%)	< 18.5	≥ 25 < 30	≤ 18.5 < 25	≥ 30
	12%	20%	56%	12%
BMI before delivery (%)	< 18.5	≥ 25 < 30	≤ 18.5 < 25	≥ 30
	0%	36%	16%	48%
Mass gain (kg) during pregnancy	< 12	12–18	>18 36%	
	25%	39%		

**Table 2:** Group characteristics: children up to 24 months old.

Characteristic	Age of Children (months)			
	1	6	12	24
	*N* = 81	*N* = 82	*N* = 78	*N* = 73
Gender (% male)	53.0 % (*n* = 43)	52.4% (*n* = 43)	51.3 % (*n =* 40)	47.9 % (*n =* 35)
Method of delivery (% vaginal)	35.0 % (*n =* 29)	35.4% (*n =* 30)	37.2 % (*n =* 29)	35.6 % (*n =* 26)
Antibiotics (%)	3.7 % (*n =* 3)	21.9% (*n =* 18)	41.0 % (*n =* 32)	74.0 % (*n =* 54)
Birth weight:				
≤ 15th percentile	14.8% (*n =* 12)	12.2% (*n =* 10)	12.8% (*n =* 10)	12.3% (*n =* 9)
≥ 85th percentile	13.6% (*n =* 11)	13.4% (*n =* 11)	12.8% (*n =* 10)	12.3% (*n =* 9)
Mass (kg) at each age:				
(mean + SD)	4.537 ± 0.611	7.953 ± 1.095	10.131 ± 1.129	12.91 ± 1.642
(range)	(2.780, 5.970)	(6, 10)	(7.89, 13)	(10, 17)
≤ 15th percentile	12.3% (*n =* 10)	20.7 % (*n =* 17)	1.3 % (*n =* 1)	2.7 % (*n =* 2)
15–85th percentile	64.2 % (*n =* 52)	54.9 % (*n =* 45)	64.1 % (*n =* 50)	61.6 % (*n =* 45)
≥ 85th percentile	24.7 % (*n =* 20)	24.4 % (*n =* 20)	34.6 % (*n =* 27)	35.6 % (*n =* 26)
Feeding method (% artificial)	18.5 % (*n =* 15)	48.8 % (*n =* 40)	83.3 % (*n =* 65)	95.9 % (*n =* 70)

## Supplementary Materials

The following are available online at https://www.mdpi.com/2077-0383/9/3/777/s1 Figure S1: Zonulin concentrations over time with feeding type; Figure S2: Calprotectin concentrations over time with feeding type. Table S1: Zonulin concentrations in stool at different times after birth; Table S2: Calprotectin concentrations in stool at different times after birth; Table S3: The effects of antibiotic therapy during pregnancy on zonulin concentrations; Table S4: Influence of method of delivery on zonulin concentrations; Table S5: The effect of delivery method and antibiotic therapy at delivery on zonulin and calprotectin concentrations; Table S6: Influence of maternal BMI increase during pregnancy on zonulin and calprotectin concentrations; Table S7: Effect of birth weight on zonulin and calprotectin concentrations; Table S8: Influence of children’s body weights on zonulin and calprotectin concentrations, divided according to the 15th percentile; Table S9: Influence of children’s body weight on zonulin and calprotectin concentrations, divided according to the 85th percentile; Table S10: Effect of body weight on zonulin and calprotectin concentrations: comparison of ≤ 15th percentile with ≥ 85th percentile; Table S11: Influence of children’s antibiotic therapy on zonulin and calprotectin concentrations.
