# Supplementary material for: Analysis of Faecal Zonulin and Calprotectin Concentrations in Healthy Children During the First Two Years of Life. An Observational Prospective Cohort Study
Source: J Clin Med. 2020 Mar 12;9(3):777. doi: 10.3390/jcm9030777 (PMC7141325; doi:10.3390/jcm9030777)
Supplement: Supplementary file 1 [file jcm-09-00777-s001.pdf]

Supplementary materials:

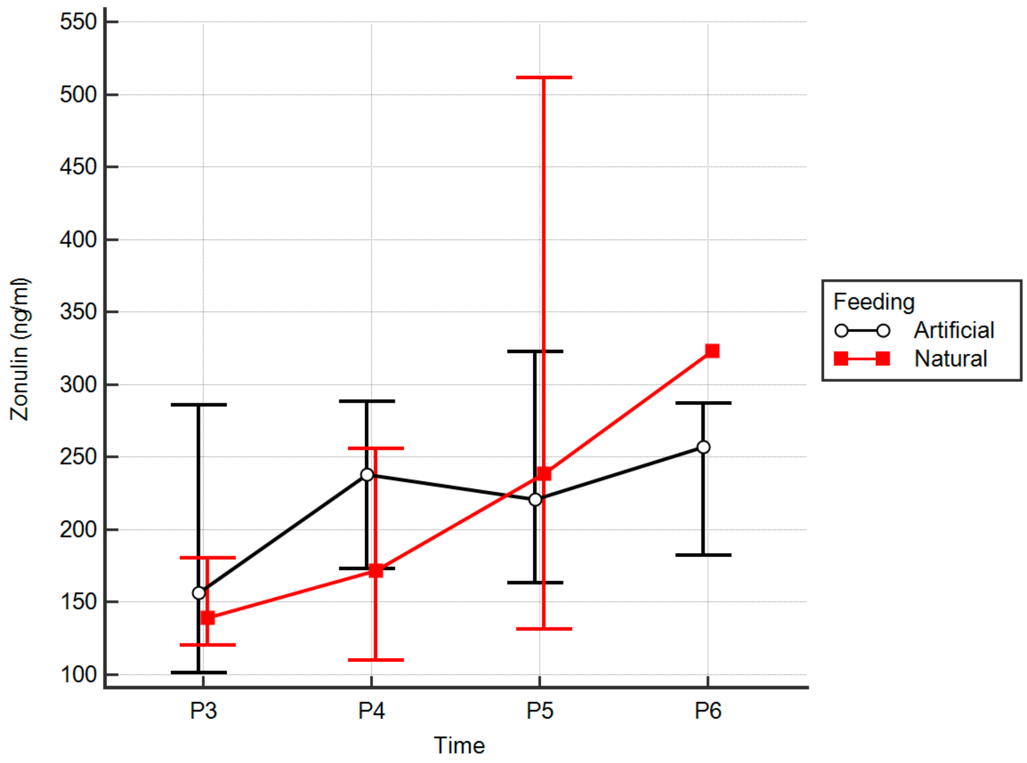

Feeding method	1st month (P3)	6th month (P4)	12th month (P5)	24th month (P6)
**Natural**	n=60	n=35	n=8	n=3
ZON Median (ng/ml) (range)	139.32 (41.58-670.03)	175.89 (43.31-721,13)	238.75 (69.07-735.55)	311.18 (279.45-367.04)
**Artificial**	n=12	n=26	n=56	n=57
ZON Median (ng/ml) (range)	156.35 (75.90-712.03)	237.98 (41.32-572.14)	220.84 (49.45-781.53)	256.90 (38.06-695.67)
**p^&^ (r)**	0.728 (-0.04)	0.164 (0.18)	-*	-*

Supplementary Figure S1. Zonulin (ZON) concentrations (median) over time with feeding type. Error bars represent 95% confidence intervals for medians. $p^{\&}$ = Mann-Whitney test comparing feeding types, r = effect size, * - statistics not performed due to small sample size.

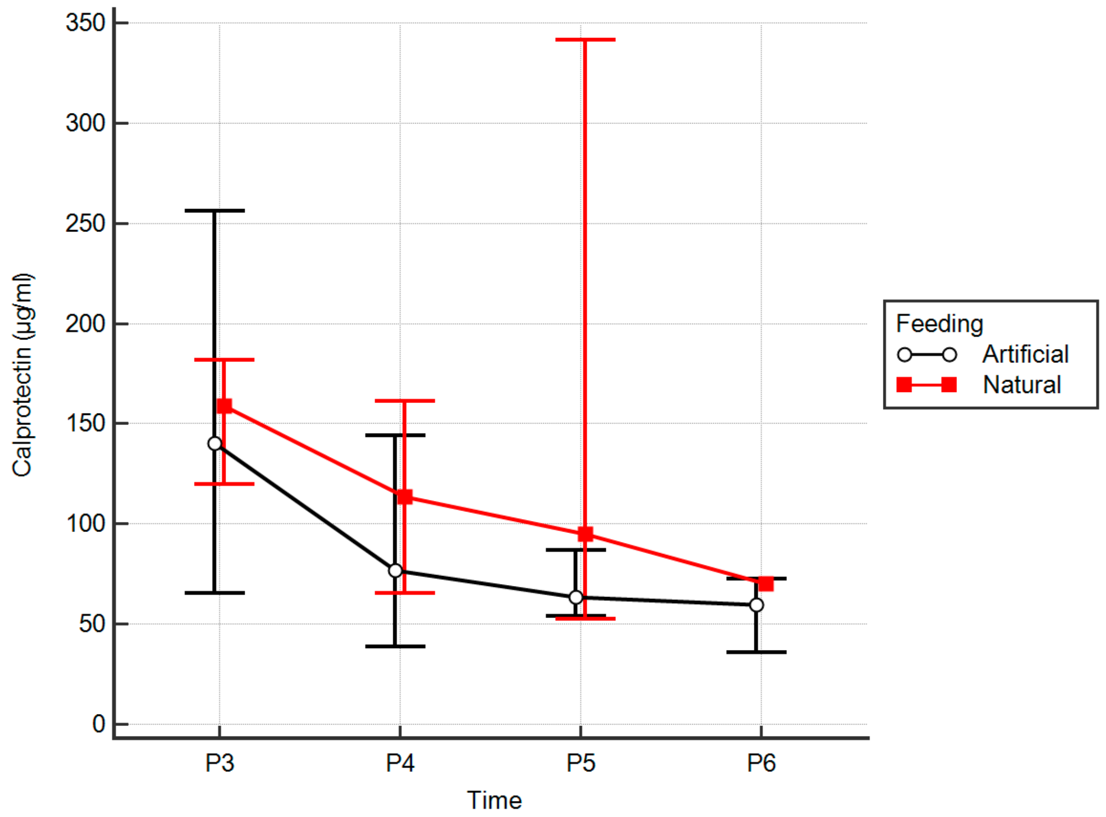

Feeding method	1st month (P3)	6th month (P4)	12th month (P5)	24th month (P6)
**Natural**	n=63	n=36	n=9	n=2
CALP Median (µg/ml) (range)	155.42 (23.51, 681.81)	113.46 (3.68, 378.90)	94.81 (18.4, 1447.15)	70.18 (53.47, 86.87)
**Artificial**	n=10	n=33	n=54	n=47
CALP Median (µg/ml) (range)	140.19 (12.62, 317.00)	76.82 (4.51, 218.89)	66.42 (3.03, 289.46)	59.55 (2.46, 495.46)
**p$^{\&}$ (r)**	0.84 (0.02)	0.094 (-0.2)	-*	-*

Supplementary Figure S2. Calprotectin (CALP) concentrations (median) over time with feeding type. Error bars represent 95% confidence intervals for medians. p$^{\&}$ = Mann-Whitney test comparing feeding types, r = effect size, * - statistics not performed due to small sample size.

Supplementary. Table. S1. Zonulin concentrations (ZON, ng/ml) in stool at different times after birth.

	Meconium*	7 days*	1 month	6 months	12 months	24 months
**Concentrations (ng/ml)**						
**ZON Median (range)**	**n=52**	**n=68**	**n=73**	**n=61**	**n=65**	**n=61**
	54.15 (1.36-700.65)	114.41 (29.38-593.72)	139. 61 (41.58-712.03)	195.67 (41.32-721.13)	223.7 (49.45-781.53)	256.9 (38.06-695.67)
**Statistical analyses (Upper-right: differences; Lower-left: correlations)**						
**Meconium***	x	**n=36** p=0.0002$^{\$}$ r=0.313	**n=38** p=0.00001$^{\$}$ r=0.71	**n=31** p=0.00021$^{\$}$ r=0.67	**n=36** p=0.000003$^{\$}$ r=0.78	**n=33** p=0.00002$^{\$}$ r=0.61

**7 days***	**n=36**<br>R=0.31<br>p=0.063	x	**n=55**<br>p=0.0756[$]<br>r=0.24	**n=45**<br>p=0.0083[$]<br>r=0.39	**n=49**<br>p=0.0007[$]<br>r=0.48	**n=44**<br>p=0.00001[$]<br>r=0.64
**1 month**	**n=38**<br>R=0.34<br>p=0.039	**n=55**<br>R=0.28<br>p= 0.041	x	**n=52**<br>p=0.0645[$]<br>r=0.26	**n=57**<br>p=0.0002[$]<br>r=0.49	**n=51**<br>p=0.00002[$]<br>r=0.58
**6 months**	**n=31**<br>R=0.07<br>p=0.7	**n=45**<br>R=0.01<br>p=0.953	**n=52**<br>R=-0.09<br>p=0.515	x	**n=48**<br>p=0.1239[$]<br>r=0.22	**n=46**<br>p=0.058[$]<br>r=0.28
**12 months**	**n=36**<br>R= -0.39<br>p=0.019	**n=49**<br>R=-0.003<br>p=0.983	**n=57**<br>R=0.05<br>p=0.701	**n=48**<br>R=0.09<br>p=0.536	x	**n=52**<br>p=0.8841[$]<br>r=0.02
**24 months**	**n=33**<br>R=0.216<br>p=0.227	**n=44**<br>R=0.253<br>p=0.09	**n=51**<br>R=0.32<br>p=0.022	**n=46**<br>R=-0.08<br>p=0.607	**n=52**<br>R=0.07<br>p=0.628	x

n- number of observations, R –correlation coefficient (Spearman's), p –statistical significance ([$]- Wilcoxon signed rank test), r – effect size; * - to better illustrate changes in ZON concentrations in the first two years of life, the table includes ZON concentrations in meconium and stool from the 7th day of life (these results were presented in our previous work [28]).

Supplementary Table. S2. Calprotectin concentrations (CALP, μg/ml) in stool at different times after birth.

	**Meconium***	**7 days***	**1 month**	**6 months**	**12 months**	**24 months**
**Concentration (μg/ml)**						
**CALP Median (range)**	**n=76**<br>154.76 (6.93-884.11)	**n=72**<br>139.12 (11.89-627.35)	**n=74**<br>149.29 (12.63-681.81)	**n=70**<br>109.28 (3.68-378.9)	**n=64**<br>74.18 (3.03-1447.15)	**n=49**<br>59.50 (2.46-495.46)
**Statistical analysis (Upper-right: differences; Lower-left: correlations)**						
**Meconium***	x	**n=65**<br>p=0.179[$]<br>r=0.17	**n=68**<br>p=0.191[$]<br>r=0.16	**n=64**<br>p=0.00002[$]<br>r=0.54	**n=58**<br>p=0.00001[$]<br>r=0.58	**n=45**<br>p=0.00001[$]<br>r=0.66
**7 days***	**n=65**<br>R=0.45<br>p=0.0002	x	**n=64**<br>p=0.840[$]<br>r=0.03	**n=59**<br>p=0.0666[$]<br>r=0.24	**n=55**<br>p=0.0001[$]<br>r=0.52	**n=43**<br>p=0.00025[$]<br>r=0.55
**1 month**	**n=68**<br>R=0.39<br>p=0.0011	**n=64**<br>R=0.4<br>p= 0.0011	x	**n=62**<br>p=0.00012[$]<br>r=0.49	**n=58**<br>p=0.000005[$]<br>r=0.61	**n=45**<br>p=0.00004[$]<br>r=0.61
**6 months**	**n=64**<br>R=0.26<br>p=0.0383	**n=59**<br>R=0.31<br>p=0.0157	**n=62**<br>R=0.33<br>p=0.0095	x	**n=57**<br>p=0.0944[$]<br>r=0.22	**n=42**<br>p=0.0651[$]<br>r=0.28
**12 months**	**n=58**<br>R=0.16<br>p=0.2213	**n=55**<br>R=0.16<br>p=0.2531	**n=58**<br>R=0.28<br>p=0.0318	**n=57**<br>R=0.54<br>p=0.00002	x	**n=42**<br>p=0.4345[$]<br>r=0.12
**24 months**	**n=45**<br>R=0.132<br>p=0.387	**n=43**<br>R=0.16<br>p=0.289	**n=45**<br>R=0.12<br>p=0.412	**n=42**<br>R=0.28<br>p=0.0744	**n=42**<br>R=0.10<br>p=0.52	x

n- number of observations, R –correlation coefficient (Spearman's), p –statistical significance ($-Wilcoxon signed rank test), r – effect size, * - to better illustrate changes in CALP concentrations in the first two years of life, the table includes CALP concentrations in meconium and stool from the 7th day of life (these results were presented in our previous work [28]).

Supplementary. Table. S3 The effects of antibiotic therapy during pregnancy on zonulin (ZON, ng/ml) concentrations.

	**Zonulin concentrations (ng/ml) with Antibiotics during pregnancy (yes or no)**		
	**Yes Median (range)**	**No Median (range)**	**p /r**
**ZON in meconium***	**n=16**	**n=36**	0.117/-0.22
	30.32 (2.13-700.65)	62.42 (1.36-309.2)	
**ZON 7 days***	**n=21**	**n=47**	0.248/-0.14
	105.23 (55.03-339.46)	125.2 (29.38-593.72)	
**ZON 1 month**	**n=18**	**n=55**	0.622/-0.06
	124.93 (44.01-346.7)	147.14 (41.58-712.03)	
**ZON 6 months**	**n=16**	**n=45**	0.309/-0.13
	192.81 (50.73-445.67)	195.67 (41.32-721.13)	
**ZON 12 months**	**n=18**	**n=47**	0.582/0.07
	289 (95.68-763.98)	217.98 (49.45-781.53)	
**ZON 24 months**	**n=16**	**n=45**	0.168/-0.19
	180.18 (38.06-610.46)	270.41 (52.72-695.67)	

n- number of observations, p – statistical significance , r – effect size.* - to better illustrate changes in ZON concentrations in the first two years of life, the table includes ZON concentrations in meconium and stool from the 7th day of life (these results were presented in our previous work [28]).

Supplementary. Table. S4. Influence of method of delivery on zonulin concentrations (ZON, ng/ml).

	**Vaginal birth Median (range)**	**Caesarean section Median (range)**	**p/r**
**ZON in meconium***	**n=24**	**n=28**	0.283/-0.15
	32.59 (1.93-309.2)	60.63 (1.36-700.65)	
**ZON 7 days***	**n=29**	**n=39**	0.002/-0.38
	92.49 (29.38-323.93)	142.46 (50.97-593.72)	
**ZON 1 month**	**n=26**	**n=47**	0.561/-0.07
	138.36 (42.4-712.03)	165.02 (41.58-670.03)	
**ZON 6 months**	**n=24**	**n=37**	0.877/-0.02
	215.47 (43.31-494.1)	188.55 (41.32-721.13)	
**ZON 12 months**	**n=23**	**n=42**	0.656/-0.06
	191.79 (104.9-735.55)	250.14 (49.45-781.53)	
**ZON 24 months**	**n=20**	**n=41**	0.987/0.04
	256.9 (62.97-688.55)	264.82 (38.06-695.67)	

n- number of observations, p – statistical significance , r – effect size. * - to better illustrate changes in zonulin concentrations in the first two years of life, the table includes ZON concentrations in meconium and stool from the 7th day of life (these results were presented in our previous work [28]).

Supplementary. Table. S5. The effect of delivery method and antibiotic therapy at delivery on zonulin (ZON, ng/ml) and calprotectin (CALP, µg/ml) concentrations.

	Marker concentrations with vaginal birth: with (NAMP) or without antibiotics (N) or caesarean section (CC).			
	N Median (range)	NAMP Median (range)	CC Median (range)	p /r
ZON in meconium*	n=10	n=10	n=28	0.268/0.06
	81.13 (1.93-181.82)	23.01 (3.77-309.20)	60.63 (1.36-700.65)	
ZON 7 days*	n=12	n=13	n=39	0.005/0.17
	85.70 (52.84-173.40)	88.24 (29.38-323.93)	142.46 (50.97-593.72) 593.72)	
ZON 1 month	n=13	n=10	n=47	0.08/0.07
	180.52 (52.12-712.03)	102.37 (43.4-218.25)	165.02 (41.58-670.03)	
ZON 6 months	n=12	n=9	n=37	0.995/0.00
	237.98 (43.31-378.27)	175.89 (85.76-494.1)	188.55 (41.32-721.13)	
ZON 12 months	n=10	n=10	n=42	0.406/0.03
	209.6 (127.21-735.55)	151.72 (104.09-648.88)	250.14 (49.45-781.53)	
ZON 24 months	n=8	n=9	n=41	0.747/0.02
	234.66 (62.97-524.11)	234.66 (146.61-524.11)	264.82 (38.06-695.67)	
CALP in meconium*	n=12	n=12	n=48	0.734/0.009
	128.54 (32.39-570.49)	157.59 (20.28-737.25)	169.73 (6.93-884.11)	
CALP 7 days*	n=15	n=11	n=42	0.333/0.03
	64.15 (35.76-530.60)	165.63 (56.38-235.59)	139.12 (11.89-627.35)	
CALP 1 month	n=14	n=10	n=47	0.394/0.03
	116.61 (23.52-681.81)	172 (51.45-297.73)	155.49 (12.63-608.67)	
CALP 6 months	n=12	n=10	n=46	0.045/0.09
	146.75 (16.95-327.98)	161.87 (6.68-269.79)	65.29 (3.68-378.9)	
CALP 12 months	n=12	n=9	n=40	0.258/0.05
	54.02 (3.03-1447.15)	126.83 (18.4-354.68)	72.37 (9.41-264.17)	
CALP 24 months	n=9	n=7	n=30	0.957/0.00
	60.98 (10.53-194.7)	38.51 (23.83-149.89)	54.41 (2.46-495.46)	

***ZON 7 days: post-hoc analyses:** N vs. CC p= 0.027, NAMP vs. CC p=0.031, N vs. NAMP p=1.0 **CALP 6 months: post-hoc analyses:** N vs. CC p = 0.129, NAMP vs. CC p = 0.208, N vs. NAMP p = 1.0; N=Natural birth without antibiotics, NAMP=Natural birth with prophylactic administration of ampicillin, CC=Caesarean section with application of cefazolin. n- number of observations, p – statistical significance, r – effect size. * - to better illustrate changes in zonulin and calprotectin concentrations in the first two years of life, the table includes ZON and CALP concentrations in meconium and stool from the 7th day of life (these results were presented in our previous work [28]).

Supplementary Table. S6. Influence of maternal BMI increase during pregnancy on zonulin (ZON, ng/ml) and calprotectin (CALP, µg/ml) concentrations.

	Increase in BMI (kg/m²)		
	>5.7 Median (range)	<=5.7 Median (range)	p/r
ZON Meconium*	n=24	n=27	0.210/-0.18
	47.38 (1.36-309.20)	63.53 (1.93-700.65)	
ZON 7 days*	n=28	n=38	0.215/-0.15
	107.30 (50.97-505.51)	133.51 (29.38-593.72)	
ZON 1 month	n=37	n=33	0.911/-0.01
	139.03 (41.58-712.03)	171.26 (43.40-670.03)	

**ZON 6 months**	**n=28**	**n=31**	0.320/-0.13
	183.85 (50.73-445.67)	217.54 (41.32-721.13)	
**ZON 12 months**	**n=32**	**n=30**	0.049/0.25
	315.19 (69.07-781.53)	181.53 (49.45-763.98)	
**ZON 24 months**	**n=29**	**n=28**	0.52/-0.08
	256.90 (38.06-695.67)	286.21 (52.72-688.55)	
**CALP Meconium***	**n=32**	**n=41**	0.523/0.07
	170.52 (11.74-884.11)	143.27 (6.93-737.25)	
**CALP 7 days ***	**n=32**	**n=37**	0.030/0.26
	153.35 (26.58-571.09)	113.67 (23.27-627.35)	
**CALP 1 month**	**n=34**	**n=37**	0.752/0.04
	152.83 (24.31-681.81)	134.29 (12.63-317.01)	
**CALP 6 months**	**n=33**	**n=35**	0.854/-0.02
	112.36 (4.20-378.90)	91.69 (3.68-327.98)	
**CALP 12 months**	**n=31**	**n=30**	0.920/0.01
	87.03 (9.41-173.54)	60.59 (3.03-1447.15)	
**CALP 24 months**	**n=24**	**n=22**	0.28/0.16
	62.69 (2.46-495.46)	39.79 (5.63-194.70)	

n- number of observations, p – statistical significance, r – effect size.* - to better illustrate changes in zonulin and calprotectin concentrations in the first two years of life, the table includes ZON and CALP concentrations in meconium and stool from the 7th day of life (these results were presented in our previous work [28]).

Supplementary Table S7. Effect of birth weight on zonulin (ZON, ng/ml) and calprotectin (CALP, μg/ml) concentrations.

	**Birth weight (kg)**		
	**≤ 15th percentile Median (range)**	**≥ 85th percentile Median (range)**	**p/r**
**ZON Meconium***	**n=9**	**n=6**	0.238/-0.30
	28.91 (8.14-148.24)	87.95 (7.40-122.11)	
**ZON 7 days***	**n=11**	**n=7**	0.085/-0.41
	78.91 (55.03-593.72)	114.50 (88.24-505.51)	
**ZON 1 month**	**n=9**	**n=9**	0.659/-0.10
	147.14 (41.58-712.03)	206.48 (60.62-660.63)	
**ZON 6 months**	**n=6**	**n=9**	0.263/0.29
	299.32 (92.09-403.99)	195.67 (64.61-445.67)	
**ZON 12 months**	**n=9**	**n=8**	0.312/-0.25
	157.54 (69.07-763.98)	287.99 (138.31-781.53)	
**ZON 24 months**	**n=7**	**n=9**	0.244/0.29
	270.41 (157.92-688.55)	153.39 (38.06-695.67)	
**CALP Meconium***	**n=11**	**n=10**	0.699/-0.08
	161.35 (11.43-733.43)	196.29 (11.74-884.11)	
**CALP 7 days ***	**n=10**	**n=10**	0.162/-0.31
	108.52 (35.76-178.07)	172.04 (49.76-518.81)	
**CALP 1 month**	**n=11**	**n=7**	0.587/0.13
	177.02 (12.63-301.48)	100.97 (29.01-221.43)	
**CALP 6 months**	**n=10**	**n=10**	0.678/0.09
	118.46 (29.90-195.92)	84.58 (4.20-378.90)	
**CALP 12 months**	**n=8**	**n=9**	0.136/0.36

	86.66 (30.99-200.68)	46.67 (5.64-173.54)	
**CALP 24 months**	**n=5**	**n=8**	0.714/0.10
	115.95 (23.83-191.57)	73.80 (19.24-495.46)	

n- number of observations, p – statistical significance of correlation, r – effect size.* - to better illustrate changes in ZON and CALP concentrations in the first two years of life, the table includes ZON and CALP concentrations in meconium and stool from the 7th day of life (these results were presented in our previous work [28]).

Supplementary. Table. S8. Influence of childrens' body weights on zonulin (ZON, ng/ml) and calprotectin (CALP, μg/ml) concentrations, divided according to the 15th percentile.

	**Body mass (kg)**		
	**≤ 15th percentile Median (range)**	**>15th percentile Median (range)**	**p/r**
**ZON 1 month**	**n=8**	**n=65**	0.207/-0.15
	196.00 (75.90-581.19)	137.69 (41.58-712.03)	
**ZON 6 months**	**n=14**	**n=47**	0.509/0.08
	166.26 (43.31-432.83)	217.54 (41.32-721.13)	
**ZON 12 months**	**n=1**	**n=64**	-*
	763.98	220.84 (49.45-781.53)	
**ZON 24 months**	**n=1**	**n=59**	-*
	311.21	256.9 (38.06-695.67)	
**CALP 1 month**	**n=10**	**n=64**	0.981/0.00
	142.48 (12.63-681.81)	149.29 (21.84-608.67)	
**CALP 6 months**	**n=15**	**n=55**	0.241/-0.14
	133.83 (28.80-378.90)	103.68 (3.68-327.98)	
**CALP 12 months**	**n=1**	**n=63**	-*
	81.32	72.81 (3.03-1447.15)	
**CALP 24 months**	**n=1**	**n=48**	-*
	38.51	60.26 (2.46-495.46)	

n- number of observations, p – statistical significance of correlation, r – effect size, * - statistics not performed due to small sample size.

Supplementary Table. S9. Influence of children's body weights on zonulin (ZON, ng/ml) and calprotectin (CALP, μg/ml) concentrations, divided according to the 85th percentile.

	**Body mass**		
	**<85th percentile Median (range)**	**≥ 85th percentile Median (range)**	**p/r**
**ZON 1 month**	**n=56**	**n=17**	0.917/-0.01
	139.32 (41.58-712.03)	140.84 (48.67-660.63)	
**ZON 6 months**	**n=48**	**n=13**	0.449/0.10
	187.38 (41.32-572.14)	240.70 (92.33-721.13)	
**ZON 12 months**	**n=42**	**n=23**	0.300/0.13
	195.02 (80.78-763.98)	290.28 (49.45-781.53)	

**ZON 24 months**	**n=37**<br>227.19<br>(38.06-688.55)	**n=23**<br>270.41<br>(62.97-695.67)	0.53/0.08
**CALP 1 month**	**n=55**<br>152.49<br>(12.63-681.81)	**n=19**<br>142.13<br>(29.01-608.67)	0.473/0.08
**CALP 6 months**	**n=54**<br>113.46<br>(4.20-378.90)	**n=16**<br>96.01<br>(3.68-296.80)	0.994/0.00
**CALP 12 months**	**n=44**<br>66.42<br>(3.03-1447.15)	**n=20**<br>83.71<br>(11.69-289.46)	0.648/0.06
**CALP 24 months**	**n=30**<br>55.13<br>(5.63-495.46)	**n=19**<br>59.55<br>(2.46-194.7)	0.91/-0.01

n- number of observations, p – statistical significance of correlation, r – effect size.

Supplementary. Table. S10. Effect of body weight on zonulin (ZON, ng/ml) and calprotectin (CALP, μg/ml) concentrations: comparison of ≤15th percentile with ≥85th percentile.

	**Body mass**		
	**≤15th percentile Median (range)**	**≥85th percentile Median (range)**	**p/r**
**ZON 1 month**	**n=8**<br>196.00<br>(75.90-581.19)	**n=17**<br>140.84<br>(48.67-660.63)	0.367/0.18
**ZON 6 months**	**n=14**<br>166.26<br>(43.31-432.83)	**n=13**<br>240.70<br>(92.33-721.13)	0.482/0.14
**ZON 12 months**	**n=1**<br>763.98	**n=23**<br>290.28<br>(49.45-781.53)	-
**ZON 24 months**	**n=1**<br>311.21	**n=23**<br>270.41<br>(62.97-695.67)	-
**CALP 1 month**	**n=10**<br>142.48<br>(12.63-681.81)	**n=19**<br>142.13<br>(29.01-608.67)	0.697/-0.07
**CALP 6 months**	**n=15**<br>133.83<br>(28.80-378.90)	**n=16**<br>96.01<br>(3.68-296.80)	0.489/-0.12
**CALP 12 months**	**n=1**<br>81.32	**n=20**<br>83.71<br>(11.69-289.46)	-*
**CALP 24 months**	**n=1**<br>38.51	**n=19**<br>59.55<br>(2.46-194.7)	-*

n- number of observations, p – statistical significance of correlation, r – effect size, * - statistics not performed due to small sample size.

Supplementary Table 11. Influence of children's antibiotic therapy on zonulin (ZON, ng/ml) and calprotectin (CALP, μg/ml) concentrations.

	Stage:	1 month (P3)	6 months (P4)	12 months (P5)	24 months (P6)
**ZON (ng/ml)**	**ABO YES**	n=3	n=10	n=15	n=33
	**Median (range)**	123.96 (108.39, 355.63)	260.08 (128.00, 572.14)	191.79 (95.68, 659.90)	256.90 (38.06, 695.67)
	**ABO NO**	n=70	n=51	n=50	n=27
	**Median (range)**	140.22 (12.63, 552.14)	188.55 (3.68, 327.97)	223.70 (3.03, 1447.15)	264.82 (2.46, 194.7)
	**$p^{\&}$/r**	-*	0.18 (-0.15)	0.72 (0.13)	0.98 (0.04)
**CALP (μg/ml)**	**ABO YES**	n=3	n=12	n=14	n=27
	**Median (range)**	78.59 (23.51, 83.46)	148.56 (33.19, 218.98)	60.72 (29.03, 256.94)	62.62 (2.46, 495.46)
	**ABO NO**	n=71	n=58	n=50	n=22
	**Median (range)**	153.16 (12.62, 681.81)	90.12 (3.68, 378.90)	80.20 (3.03, 1447.15)	44.42 (5.62, 149.51)
	**$p^{\&}$/(r)**	-*	0.24 (-0.16)	0.78 (0.001)	0.29 (-0.04)

ABO = antibiotic therapy; n = number of subjects; p = statistical significance; r = effect size; *- statistics not performed due to small sample size.
